# Performance of Automated Attenuation Measurements at Identifying Large Vessel Occlusion Stroke on CT Angiography

**DOI:** 10.1007/s00062-020-00956-5

**Published:** 2020-09-16

**Authors:** Paul Reidler, Lena Stueckelschweiger, Daniel Puhr-Westerheide, Katharina Feil, Lars Kellert, Konstantinos Dimitriadis, Steffen Tiedt, Moriz Herzberg, Jan Rémi, Thomas Liebig, Matthias P. Fabritius, Wolfgang G. Kunz

**Affiliations:** 1 Department of Radiology, University Hospital, LMU Munich, Marchioninistr. 15, 81377 Munich, Germany; 2 Department of Neurology, University Hospital, LMU Munich, Marchioninistr. 15, 81377 Munich, Germany; 3 German Center for Vertigo and Balance Disorders, LMU Munich, Marchioninistr. 15, 81377 Munich, Germany; 4 Institute for Stroke and Dementia Research, LMU Munich, Feodor-Lynen-Str. 17, 81377 Munich, Germany; 5 Department of Neuroradiology, University Hospital, LMU Munich, Marchioninistr. 15, 81377 Munich, Germany

**Keywords:** Stroke, Perfusion, Stroke triage, Automated analysis, Thrombectomy

## Abstract

**Purpose:**

Computed tomography angiography (CTA) is routinely used to detect large-vessel occlusion (LVO) in patients with suspected acute ischemic stroke; however, visual analysis is time consuming and prone to error. To evaluate solutions to support imaging triage, we tested performance of automated analysis of CTA source images (CTASI) at identifying patients with LVO.

**Methods:**

Stroke patients with LVO were selected from a prospectively acquired cohort. A control group was selected from consecutive patients with clinically suspected stroke without signs of ischemia on CT perfusion (CTP) or infarct on follow-up. Software-based automated segmentation and Hounsfield unit (HU) measurements were performed on CTASI for all regions of the Alberta Stroke Program Early CT score (ASPECTS). We derived different parameters from raw measurements and analyzed their performance to identify patients with LVO using receiver operating characteristic curve analysis.

**Results:**

The retrospective analysis included 145 patients, 79 patients with LVO stroke and 66 patients without stroke. The parameters hemispheric asymmetry ratio (AR), ratio between highest and lowest regional AR and M2-territory AR produced area under the curve (AUC) values from 0.95–0.97 (all *p* < 0.001) for detecting presence of LVO in the total population. Resulting sensitivity (sens)/specificity (spec) defined by the Youden index were 0.87/0.97–0.99. Maximum sens/spec defined by the specificity threshold ≥0.70 were 0.91–0.96/0.77–0.83. Performance in a small number of patients with isolated M2 occlusion was lower (AUC: 0.72–0.85).

**Conclusion:**

Automated attenuation measurements on CTASI identify proximal LVO stroke patients with high sensitivity and specificity. This technique can aid in accurate and timely patient selection for thrombectomy, especially in primary stroke centers without CTP capacity.

**Electronic supplementary material:**

The online version of this article (10.1007/s00062-020-00956-5) contains supplementary material, which is available to authorized users.

## Introduction

Detection of large vessel occlusion (LVO) in patients with suspected ischemic stroke is the most critical diagnostic challenge in acute stroke imaging. LVO stroke contributes to around 60% of stroke disability and 90% of stroke mortality, although only representing 24–38% of cases [1, 2]. Since the positive endovascular thrombectomy (EVT) trials of 2015 [3] and the recent studies on EVT beyond 6 h [4, 5], LVO patients require this highly effective treatment to ensure the best possible clinical outcome.

In most stroke treating facilities, single-phase computed tomography angiography (CTA) is the primary imaging modality to detect LVO and prompt further treatment or transfer to comprehensive stroke centers [6]. The latter is a common scenario in primary stroke centers (PSC) with often limited imaging capacities and imaging interpretation by less experienced readers compared to high-volume centers [6, 7].

Considering the literature, visual detection rate of LVO on CTA varies considerably with reported rates of missed LVO around 20% [8, 9]. While multiphase CTA and CT perfusion (CTP) provide added value for LVO detection, they are not universally available, especially in smaller stroke treating facilities [6, 9, 10]. Therefore, improving diagnostic confidence at single-phase CTA could ensure correct and timely stroke triage, for example to decide patient transfer.

In spite of encouraging results on CTA hypoattenuation to detect ischemia, this imaging biomarker is currently underused and not recommended in the latest guidelines [11]; however, hypoattenuation on CTA has frequently been shown to present substantial overlap with highly sensitive maps derived from CTP [12]. Combined with automated assessment, hypoattenuation on CTA therefore has the potential to aid as reader-independent, sensitive tool for ischemia detection.

To address the clinical value of automated attenuation analysis in single-phase CTA data, we evaluated its performance in detecting stroke patients with LVO of the anterior circulation and consequent ischemia in the medial cerebral artery (MCA) territory among a population of patients with suspected ischemic stroke.

## Material and Methods

### Study Design and Population

The retrospective study was approved by the institutional review board according to the Declaration of Helsinki of 2013 and requirement for written informed consent was waived. Patients with acute ischemic stroke due to anterior circulation large vessel occlusion were selected out of a consecutive cohort of 274 patients who were prospectively enrolled in the German Stroke Registry (clinicaltrials.gov identifier: NCT03356392). All patients were directly admitted to our institution and treated with EVT between 2015 and 2017. In total, we selected 79 LVO patients.

We included patients with:

1. internal carotid artery, M1 or M2 segment artery occlusion,
2. complete noncontrast CT, single-phase CT angiography, and CTP imaging data.

We excluded patients with:

1. prior ischemia or intracranial mass, to ensure unbiased measurement of HU values,
2. pathology of the posterior circulation,
3. non-diagnostic imaging data.

From a consecutive cohort of 664 patients between 2015 and 2016 we further selected patients who underwent full imaging assessment for suspected stroke but did not present signs of ischemia at admission or infarction at follow-up to build a stroke negative control group. Otherwise the same exclusion criteria were applied as before. This led to the inclusion of 66 patients.

All 79 LVO stroke patients were previously reported in a study on automated attenuation measurements in ASPECTS regions on noncontrast CT [13]. The previous article dealt with the classification of CTP-based criteria for late time window thrombectomy [5] on noncontrast CT data, whereas the current study uses CTA data to classify the presence of large-vessel occlusion stroke. Another study incorporating only the 79 LVO patients analyzed the overlap of CTA attenuation measurements with acute and final stroke morphology (under review). The 66 patients of the stroke negative cohort have not been previously reported.

A detailed flow-chart of patient selection is provided in Figure I of the online supplemental material.

### Image Analysis

Noncontrast CT, CTA and CTP were performed using SOMATOM Definition Force, AS+ and Flash scanners (Siemens Healthineers, Forchheim, Germany). The CTP data were processed using the manufacturer’s software (syngo Neuro Perfusion CT, Siemens Healthineers) to generate perfusion maps.

For CTA intravenous administration of 50 mL iodinated contrast medium was followed by a saline chaser of 40 mL, each with a flow rate of 5 mL/s. Imaging was performed in a single sweep from the aortic arch to the vertex with a bolus trigger of 100 HU in the aortic trunk. Tube voltage was 120 kV (SOMATOM Force, Flash) or 80 kV (SOMATOM AS+) and tube current modulation (CareDose) was used. Collimation was 0.6 mm.

The ASPECTS was determined by two blinded readers as described in previous studies [13]. Manual segmentation of total ischemic volume on cerebral blood flow (CBF) maps, ischemic core volume on cerebral blood volume (CBV) maps and final infarction on follow-up CT or MRI were performed using commercial software (OsiriX v. 8.0.2, Pixmeo, Bernex, Switzerland 2017). Final infarction was determined on follow-up imaging at CT or MRI.

### Automated Analysis of Tissue Attenuation on CTA Source Images

We used a software prototype (Syngo Via Frontier ASPECTS, Siemens Healthineers) to analyze attenuation values in ASPECTS regions at CTA [13]. The prototype performs segmentation of all 10 ASPECTS regions on CT data using a probabilistic atlas and calculates the mean attenuation in Hounsfield Units (HU) for each region.

To establish a parameter which does not require a priori information of stroke laterality, we calculated the asymmetry ratio (AR), which was defined as the ratio of the quotients right by left and left by right regional HU, using the larger value as denominator. As we assume that patients without ischemia present similar values across hemispheres the AR should be around 1, while having lower values in stroke patients. The AR was calculated for each of the 10 ASPECTS regions. From regional AR values we derived the CTA hemispheric AR as average AR of all 10 ASPECTS regions. To represent the maximum AR difference among all regions in a subject, we calculated the ratio of the outer limits of an individual’s regional values as CTA min/max AR ratio (lowest regional AR/highest regional AR). To find values sensible for M2 MCA occlusion the CTA M2 territory asymmetry ratio was defined as average AR of the ASPECTS regions insula, M2, M3, M5 and M6, which constitute the largest portion of the vascular MCA M2 segment territory. For isolated M2 occlusions we separately analyzed the CTA ASPECTS M5 region AR, as the M5 region contains the motor cortex and information about involvement can therefore aid clinical decision making in distal occlusions. No impact of microvascular or age-related white matter changes on the presented parameters was found in linear regression analysis displayed in supplemental Table I.

### Statistical Analysis

Analyses were performed in SPSS Statistics 23 (IBM, Armonk NY, USA 2016, commercial software), MedCalc version 18.10.2 (MedCalc Software, Ostend, Belgium, 2018, commercial software) and R version 3.6.3 (R Foundation for Statistical Computing, Vienna Austria). All metric and ordinal variables are reported as median (interquartile range, IQR). Categorical variables are presented as number and percentage. Receiver operating characteristic (ROC) analyses using exact binomial confidence intervals (CI) compared the diagnostic performance of parameters and area under the curve (AUC) values were calculated. Cut-off values were determined using maximization of the Youden index. To reflect the clinical importance of a high sensitivity we further determined cut-off values with maximum sensitivity at a given specificity threshold of ≥0.70. To calculate the corresponding positive and negative predictive values (PPV, NPV) we assumed a 30% rate of overall LVO patients and 4% rate of M2 occlusion patients at stroke centers as found in the literature [1, 14].

## Results

### Patient Characteristics and CTASI Attenuation Measurements

In this study 79 patients with LVO stroke were included (37 female, 42 male, median age 76 years, IQR 62–82 years). Location of the most proximal occlusion was ICA in 29.1%, M1 segment in 62.0% and M2 segment in 8.9%. A total of 66 patients without ischemia on admission imaging or infarct on follow-up were included as the stroke negative group. Most frequent diagnoses in the stroke negative group were transient ischemic attack with 46% and epilepsy with 24%. Patients in the stroke negative group presented at a younger age (median: 76 vs. 68 years, *p* = 0.04). Detailed patient characteristics are displayed in Table 1.

**Table 1:** Characteristics of the study population

	LVO stroke patients (*N* = 79)		Stroke negative patients (*N* = 66)	
**Patient data**				
*Male sex*	42	(53.2%)	27	(40.9%)
*Female sex*	37	(46.8%)	39	(59.1%)
*Median age *(years)	76	(64–82)	69	(57–78)*
Male study population	73	(63–82)	69	(53–82)
Female study population	79	(74–84)	69	(60–77)*
**Treatment data**				
Intravenous thrombolysis	50	(63%)	11	(17%)*
Endovascular thrombectomy	79	(100%)	0	(0%)*
–	**Most proximal occlusion location**		**Final diagnosis**	
–	ICA	(49.4%)	TIA	(45.5%)
	M1	(88.6%)	Epilepsy	(24.2%)
	M2	(25.3%)	Vertigo disorders	(12.1%)
			Unknown	(10.6%)
			Electrolyte derailment	(4.6%)
			Migraine	(3.0%)
**Imaging data**				
Noncontrast CT-ASPECTS	8	(8–10)	10	(10–10)*
Hypoperfusion volume	143	(108–196)	NA	
Ischemic core volume	17	(9–46)	NA	
Mismatch volume	112	(70–151)	NA	
Final infarction volume	19	(6–91)	NA	
**Automated CTA analysis parameters**				
CTA hemispheric AR	0.831	(0.788–0.888)	0.977	(0.958–0.990)*
CTA min/max AR ratio	0.799	(0.751–0.844)	0.941	(0.919–0.956)*
CTA M2 territory AR	0.830	(0.771–0.893)	0.977	(0.947–0.988)*

Values presented are count (percentage) for categorical and median (interquartile range) for ordinal or continuous variables. All volumes are presented in mL

*ASPECTS* Alberta Stroke Program Early CT Score, *ICA* internal carotid artery, *LVO* large vessel occlusion, *MCA* middle cerebral artery, *M1* M1 segment of the MCA, *M2* M2 segment of the MCA, *NIHSS* National Institutes of Health Stroke Scale, *AR* asymmetry ratio, *CTA* CT angiography

*Indicates statistical significance with *P* value <0.05

The selected attenuation parameters CTA hemispheric AR, CTA min/max AR ratio, and M2 territory AR, presented highly significant differences between LVO and control patients with all *p* < 0.001. The parameters were distinctly distributed in both groups as displayed by the density plots in Supplemental Figure II.

### Receiver Operating Characteristic Analysis of CTASI Attenuation Measurements

**Fig. 1:**
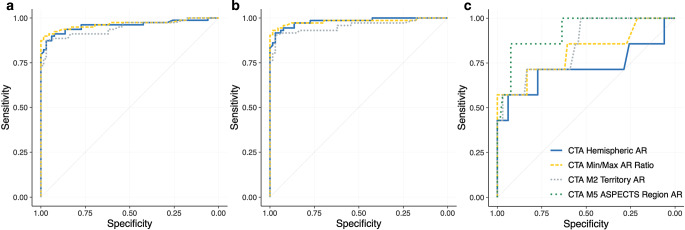
Receiver operating characteristics curves for the indicated parameters to classify LVO stroke patients and stroke negative patients. **a** Analysis in the complete cohort (79 LVO stroke patients vs. 66 stroke negative controls), **b** analysis for patients with M1 and ICA occlusion (72 LVO stroke patients vs. 66 stroke negative controls), **c** analysis for patients with M2 occlusion (7 LVO stroke patients vs. 66 stroke negative controls) (*LVO* large-vessel occlusion, *CTA* CT angiography, *M1* M1 segment of the middle cerebral artery, *M2* M2 segment of the middle cerebral artery)

The ROC curve analysis for the identification of LVO patients resulted in an AUC of 0.95–0.97 (all *p* < 0.001) for the respective parameters considering the total study cohort. An AUC of 0.95–0.98 (all *p* < 0.001) was achieved for LVO detection when comparing the stroke negative cohort and patients with ICA or M1 occlusion. In a small subsample of isolated M2 occlusions (*n* = 7) versus stroke negative patients AUCs were 0.72 (IQR: 0.60–0.82) for CTA hemispheric AR, 0.81 (IQR: 0.70–0.90) for CTA min/max AR ratio, 0.85 (IQR: 0.84–0.97) for CTA M2 territory AR and 0.92 (IQR: 0.84–0.97) for CTA ASPECTS M5 region AR. In the M2 occlusion subgroup, differences between ROC curves for LVO detection did not reach statistical significance (*p* > 0.05). Detailed results of the ROC analysis are presented in Table 2, ROC curves are provided in Fig. 1.

**Table 2:** ROC analysis for the detection of LVO patients

(*N* = 145)	AUC (95% CI)		*P* Value	Y‑Index	Y Index CP	Spec ≥70% CP
*Overall LVO Population (n* *=* *79) vs. Controls (n* *=* *66)*						
CTA Hemispheric AR	0.96	(0.91–0.98)	<0.001	0.84	0.926	0.955
CTA Min/Max AR Ratio	0.97	(0.92–0.99)	<0.001	0.88	0.810	0.889
CTA M2 Territory AR	0.95	(0.90–0.98)	<0.001	0.84	0.922	0.941
*ICA and M1 Occlusion (n* *=* *72) vs. Controls (n* *=* *66)*						
CTA Hemispheric AR	0.98	(0.94–1.00)	<0.001	0.89	0.926	0.955
CTA Min/Max AR Ratio	0.98	(0.94–1.00)	<0.001	0.92	0.810	0.955
CTA M2 Territory AR	0.95	(0.91–0.98)	<0.001	0.87	0.922	0.955
*M2 Occlusion (n* *=* *7) vs. Controls (n* *=* *66)*						
CTA Hemispheric AR	0.72	(0.60–0.82)	0.14	0.51	0.935	0.955
CTA Min/Max AR Ratio	0.81	(0.70–0.90)	0.005	0.57	0.785	0.889
CTA M2 Territory AR	0.85	(0.74–0.92)	<0.001	0.55	0.941	0.941
CTA ASPECTS M5 Region AR	0.92	(0.84–0.97)	<0.001	0.78	0.919	0.919

Cut points were determined by the Y‑Index and sensitivity threshold of ≥0.70

*ROC* indicates, receiver operating characteristics, *LVO* large vessel occlusion, *AUC* area under the curve, *CI* confidence interval, *Y‑Index* Youden Index, *Spec* specificity, *CP* cut point, *CTA* CT angiography, *AR* asymmetry ratio, *ICA* internal carotid artery, *M1* M1 segment of the middle cerebral artery, *M2* M2 segment of the middle cerebral artery, *ASPECTS* Alberta Stroke Program Early CT Score

*P* values <0.05 indicate statistical significance

### Performance Analysis of CTASI Attenuation Measurements Derived Parameters

The different cut points derived using Youden Index or sensitivity threshold ≥0.70 including their associated sensitivity/specificity and PPV/NPV are displayed in Table 3.

**Table 3:** Performance of indicated parameters for the detection of LVO stroke patients

Method/cut point	Sensitivity (95% CI) [raw #]	Specificity (95% CI) [raw #]	PPV*	NPV*
**Overall LVO Stroke Patients (*n*** **=** **79) vs. Controls (*n*** **=** **66)**				
*CTA Hemispheric AR*				
Y‑Index CP = 0.926	0.87 (0.78–0.94) [69/79]	0.97 (0.90–1.00) [64/66]	0.93 (0.76–0.98)	0.95 (0.91–0.97)
Specificity ≥0.70 CP = 0.955	0.96 (0.89–0.99) [76/79]	0.77 (0.65–0.87) [51/66]	0.65 (0.54–0.74)	0.98 (0.94–0.99)
*CTA Min/Max AR Ratio*				
Y‑Index CP = 0.810	0.90 (0.81–0.96) [71/79]	0.99 (0.92–1.00) [64/66]	0.96 (0.78–0.99)	0.96 (0.92–0.98)
Specificity ≥0.70 CP = 0.889	0.95 (0.88–0.99) [75/79]	0.83 (0.72–0.91) [55/66]	0.71 (0.59–0.81)	0.98 (0.94–0.99)
*CTA M2 Territory Asymmetry Ratio*				
Y‑Index CP = 0.922	0.87 (0.80–0.95) [69/79]	0.97 (0.90–1.00) [65/66]	0.93 (0.76–0.98)	0.95 (0.91–0.97)
Specificity ≥0.70 CP = 0.941	0.91 (0.83–0.96) [72/79]	0.83 (0.72–0.91) [55/66]	0.70 (0.58–0.80)	0.96 (0.92–0.99)
**ICA** **+** **M1-Occlusion (*n*** **=** **72) vs. Controls (*n*** **=** **66)**				
*CTA Hemispheric AR*				
Y‑Index CP = 0.926	0.92 (0.83–0.97) [66/72]	0.97 (0.90–1.00) [64/66]	0.93 (0.77–0.98)	0.96 (0.93–0.98)
Specificity ≥0.70 CP = 0.955	0.99 (0.93–1.000) [71/72]	0.77 (0.65–0.87) [51/66]	0.65 (0.54–0.74)	0.99 (0.95–1.00)
*CTA Min/Max AR Ratio*				
Y‑Index CP = 0.810	0.93 (0.85–0.97) [67/72]	0.99 (0.92–1.00) [65/66]	0.96 (0.79–1.00)	0.97 (0.93–0.99)
Specificity ≥0.70 CP = 0.955	0.97 (0.90–1.00) [70/72]	0.88 (0.78–0.95) [58/66]	0.78 (0.64–0.87)	0.99 (0.95–1.00)
*CTA M2 Territory AR*				
Y‑Index CP = 0.922	0.90 (0.83–0.97) [65/72]	0.97 (0.90–1.00) [64/66]	0.93 (0.77–0.98)	0.96 (0.93–0.98)
Specificity ≥0.70 CP = 0.955	0.93 (0.85–0.98) [67/72]	0.83 (0.72–0.91) [55/66]	0.71(0.58–0.81)	0.97 (0.92–0.99)
**M2 Occlusions (*n*** **=** **7) vs. Controls (*n*** **=** **66)**				
*CTA Hemispheric AR*				
Y‑Index CP = 0.935	0.57 (0.18–0.90) [4/7]	0.94 (0.85–0.98) [62/66]	0.28 (0.11–0.55)	0.98 (0.96–0.99)
Specificity ≥0.70 CP = 0.955	0.71 (0.20–0.96) [5/7]	0.77 (0.65–0.87) [51/66]	0.12 (0.06–0.20)	0.99 (0.95–1.00)
*CTA Min/Max AR Ratio*				
Y‑Index CP = 0.785	0.57 (0.18–0.90) [4/7]	1.0 (0.95–1.00) [66/66]	1.00 (1.00–1.00)	0.98 (0.96–0.99)
Specificity ≥0.70 CP = 0.889	0.71 (0.29–0.96) [5/7]	0.83 (0.72–0.91) [51/66]	0.15 (0.08–0.27)	0.99 (0.96–1.00)
*CTA M2 Territory AR*				
Y‑Index CP = 0.941	0.71 (0.29–0.96) [5/7]	0.83 (0.72–0.91) [55/66]	0.15 (0.08–0.27)	0.99 (0.96–1.00)
Specificity ≥0.70 (same as above)	0.71 (0.29–0.96) [5/7]	0.83 (0.72–0.91) [55/66]	0.15 (0.08–0.27)	0.99 (0.96–1.00)
*CTA M5 ASPECTS Region AR*				
Y‑Index CP = 0.919	0.86 (0.42–1.00) [6/7]	0.92 (0.83–0.98) [61/66]	0.32 (0.16–0.54)	0.99 (0.96–1.00)
Specificity ≥0.70 (same as above)	0.86 (0.42–1.00) [6/7]	0.92 (0.83–0.98) [61/66]	0.32 (0.16–0.54)	0.99 (0.96–1.00)

Performance metrics for the indicated parameters are displayed as value (95%-CI) [raw numbers]. CP were determined by the Y‑Index and sensitivity threshold of ≥0.70. To calculate PPV and NPV a rate of overall LVO stroke patients of 30% and specifically M2 occlusion patients of 4% was assumed

*LVO* indicates large-vessel occlusion, *CI* confidence interval, *PPV* positive predictive value, *NPV* negative predictive value, *Y‑Index* Youden Index, *CP* cut point, *Spec* specificity, *CTA* CT angiography, *AR* asymmetry ratio, *ICA* internal carotid artery, *M1* M1 segment of the middle cerebral artery, *M2* M2 segment of the middle cerebral artery, *ASPECTS* Alberta Stroke Program Early CT Score

**Fig. 2:**
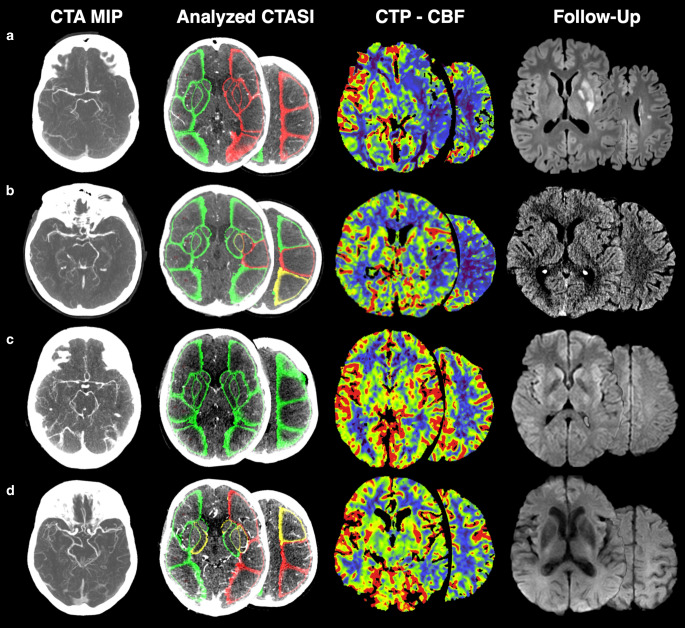
Examples of LVO classification. **a** Patient with left sided M1 occlusion. Analyzed CTASI present attenuation decrease in the affected hemisphere in the same regions as seen on CTP. This patient was correctly classified by all parameters. Follow-up MRI displays basal ganglia infarction after successful thrombectomy. **b** Patient with left sided occlusion of the proximal M2 segment. Analyzed CTASI present attenuation changes in few regions of the M2 territory. This patient was wrongly classified by CTA hemispheric AR as stroke negative but correctly classified by the other parameters CTA Min/Max AR Ratio, CTA M2 Territory Asymmetry Ratio, CTA M5 ASPECTS Region AR as LVO stroke. Follow-up CT displays slight blur of the insular ribbon after successful thrombectomy. **c** Patient who presented with right hemihypesthesia. Analyzed CTASI present equal attenuation on both hemispheres. No hypoperfusion is seen on CTP. This patient was correctly classified as LVO stroke negative by all parameters. Follow-up MRI displays no infarction. **d** Patient who presented with new dysarthria and headache. No occlusion on CTA or hypoperfusion on CTP is detectably. Analyzed CTASI present attenuation changes in few regions of left hemisphere. This patient was wrongly classified by all parameters as LVO stroke positive. Follow-up CT displays no infarction (*LVO* large-vessel occlusion, *CTA* CT angiography, *MIP* maximum intensity projection, *CTASI* CTA source images, *CTP* CT perfusion, *CBF* cerebral blood flow, *M1* M1 segment of the middle cerebral artery, *M2* M2 segment of the middle cerebral artery, *AR* asymmetry ratio)

For the overall study population, the Youden Index cut points favored specificity (0.97–0.99) over sensitivity (0.87–0.90) in our data with same values for all parameters. Using a sensitivity threshold ≥0.70 measurements achieved sensitivity between 0.91–0.96 while maintaining specificity of 0.77–0.83. Consequently, PPV for Youden Index cut points were higher (0.93–0.96 vs. 0.64–0.70) while producing a slightly lower NPV (0.95–0.96 vs. 0.96–0.98). Considering only patients with ICA or M1 occlusion produced similar results with even higher sensitivity up to 0.99. In the subsample with M2 occlusions CTA Hemispheric AR, CTA Min/Max AR ratio and CTA ASPECTS M5 Region AR achieved the same sensitivity of 0.71 at the predefined specificity threshold. CTA ASPECTS M5 Region AR presented higher sensitivity of 0.86 (IQR: 0.42–1.00) in this subsample. Assuming the rate of 4% M2 occlusion stroke patients [15], PPV reached from 0.12–0.32 and NPV from 0.98 and 0.99. Patient examples with true/false positive and true/false negative classification are provided in Fig. 2.

## Discussion

Our study presents a highly sensitive automated approach to identify patients with anterior circulation LVO stroke on single-phase CTA. The performance was especially reliable in patients with ICA or M1 occlusion while presenting lower sensitivity/specificity for isolated M2 occlusions.

Although automated analysis of CTP data has reached guideline-based recommendation status [11], CTA is still mostly the domain of visual analysis. Multiple commercial software solutions for LVO detection on CTA are already available and usually implement artificial intelligence algorithms. Vendors include ischemaView (RAPID CTA, ischemaView inc., Menlo Park, CA, USA), Viz.ai (Viz LVO, Viz.ai, San Francisco, CA, USA), Nico.lab (Stroke Viewer, nico-lab, Amsterdam, The Netherlands) or Brainomix (e-CTA, Brainomix, Oxford, UK). Reported sensitivities for LVO detection range between 0.81 and 0.97, although data are not complete for all vendors [16–20].

While other software use direct vessel analysis for LVO detection on CTA, our regional approach of attenuation analysis determines changes in tissue perfusion comparable to highly sensitive CTP maps. In contrast to the dynamic sampling of tissue attenuation during a CTP examination, CTA provides only a single measurement timepoint, in our case, determined by the bolus dynamics in the aortic arch. Notably, complex processing of 4D CTP data is necessary to produce reliable results and correct for different hemodynamics, which is not applicable for the described technique and CTA imaging in general. Nevertheless, attenuation changes on CTASI have frequently presented high sensitivity for ischemia and correlation with cerebral blood flow CTP maps [12, 21, 22]. This including our data indicate that CTA using bolus trigger seem to produce robust attenuation phases that are highly sensitive for tissue ischemia similar to CTP. Thereby, automated ischemia detection on CTA, as we examined, could complement other software solutions focusing on LVO localization in order to produce higher and more robust detection rates or even pose as surrogate for CTP.

For PSCs, where only CTA might be available as advanced imaging modality, these encouraging results indicate a benefit, as software-assisted diagnosis can elevate diagnostic confidence and support on call radiologists or neurologists without extensive experience in stroke imaging. This is an important safety aspect for CTA assessment as variability with only fair interreader agreement in LVO detection is known [10]. In a retrospective study the rate of initially missed LVO stroke was 20%, being highly associated with reader experience [8]. Especially distal occlusion seems to challenge interpreting doctors with reported sensitivities as low as 33% [9].

Relating these numbers with the proportion of LVO stroke patients at stroke centers of around 24–38% [1, 2, 23] implicates a relevant number of patients who are under risk of erroneous treatment decision. Especially in times with proven treatment effects of EVT up to 24 h and indicated effectiveness even outside the RCT criteria, the identification of treatable LVO is paramount [4, 5, 24, 25]. The range of sensitivities of our examined parameters from 0.87 to 0.99 for proximal and from 0.57 to 0.86 for distal occlusion therefore indicate a promising approach to augment visual analysis and secure correct patient selection. Notably, these results might change in studies on larger patient cohorts and in the real-life setting.

Recognizing the large number of transferred stroke patients in recent trials [26], the decision to transfer relies heavily on imaging interpretation at PSCs. Here the interpretation of CT and especially CTA examinations represent a relevant time span and cause of delay in the management of patients [7, 27]. While the additional use of CTP would significantly improve sensitivity over CTA and noncontrast CT alone even for experienced neuroradiologists [9], availability is not universal, especially among PSCs and can also cause further delay [28, 29]. Our reported sensitivity/specificity metrics would result in an overall PPV of 0.65–0.96 and NPV of 0.96–0.98. This would result in effectively ruling out LVO in suspected stroke while supporting further assessment or transfer in positive cases.

The high accuracy of the described technique encourages further studies in primary and secondary stroke centers in a prospective setting. Processing times of around 5 min using the nonoptimized software prototype also present a suitable timeframe; however this parameter was not systematically examined.

The results of our study have to be regarded in the light of the limitations. First, we only provide a limited dataset. As we present an experimental approach, the stroke negative group was highly selected without signs of ischemia on admission CTP and follow-up to ensure unbiased results. Also, patients with prior stroke were excluded in the study analysis. To further translate these methods to the clinical setting, our findings, therefore, need replication in larger prospective studies on consecutive patients including measurements of time to diagnosis and time to treatment as well as analysis of diagnostic accuracy. Notably, we strongly assume that patients with lacunar stroke without LVO, who were excluded in our study, would not greatly change the results as even CTP presents only reduced sensitivity in the acute phase [30].

Second, we only relied on a single vendor for the study using a modern CTA protocol. These protocols have been shown to overestimate the ischemic core, yet they provide a very sensitive approach to ischemia detection [21]. Therefore, translation between different platforms and protocols needs further assessment.

Third, our results are limited by the software design to perform regional attenuation measurements according to the implemented ASPECTS template. A template-free, voxel-wise approach might provide a more genuine assessment of ischemic attenuation changes on CTASI but was not available to us for this study.

In conclusion, attenuation measurements on CTASI identify proximal LVO stroke of the anterior circulation with high sensitivity and specificity and can therefore aid in accurate and timely patient selection for thrombectomy.

## Caption Electronic Supplementary Material

The Electronic supplementary material contains figures of patient selection and density plots of CT parameters as well as linear regression analysis for the influence of white matter changes
