# Supplementary material for: Performance of Automated Attenuation Measurements at Identifying Large Vessel Occlusion Stroke on CT Angiography
Source: Clin Neuroradiol. 2020 Sep 16;31(3):763–72. doi: 10.1007/s00062-020-00956-5 (PMC8463515; doi:10.1007/s00062-020-00956-5)
Supplement: Supplementary file 1 — The Electronic supplementary material contains figures of patient selection and density plots of CT parameters as well as linear regression analysis for the influence of white matter changes [file 62_2020_956_MOESM1_ESM.docx]

**Supplement**

**Supplemental Figure I. Flow chart of patient selection.** CTP indicates CT perfusion; PRES, posterior reversible encephalopathy syndrome.

**
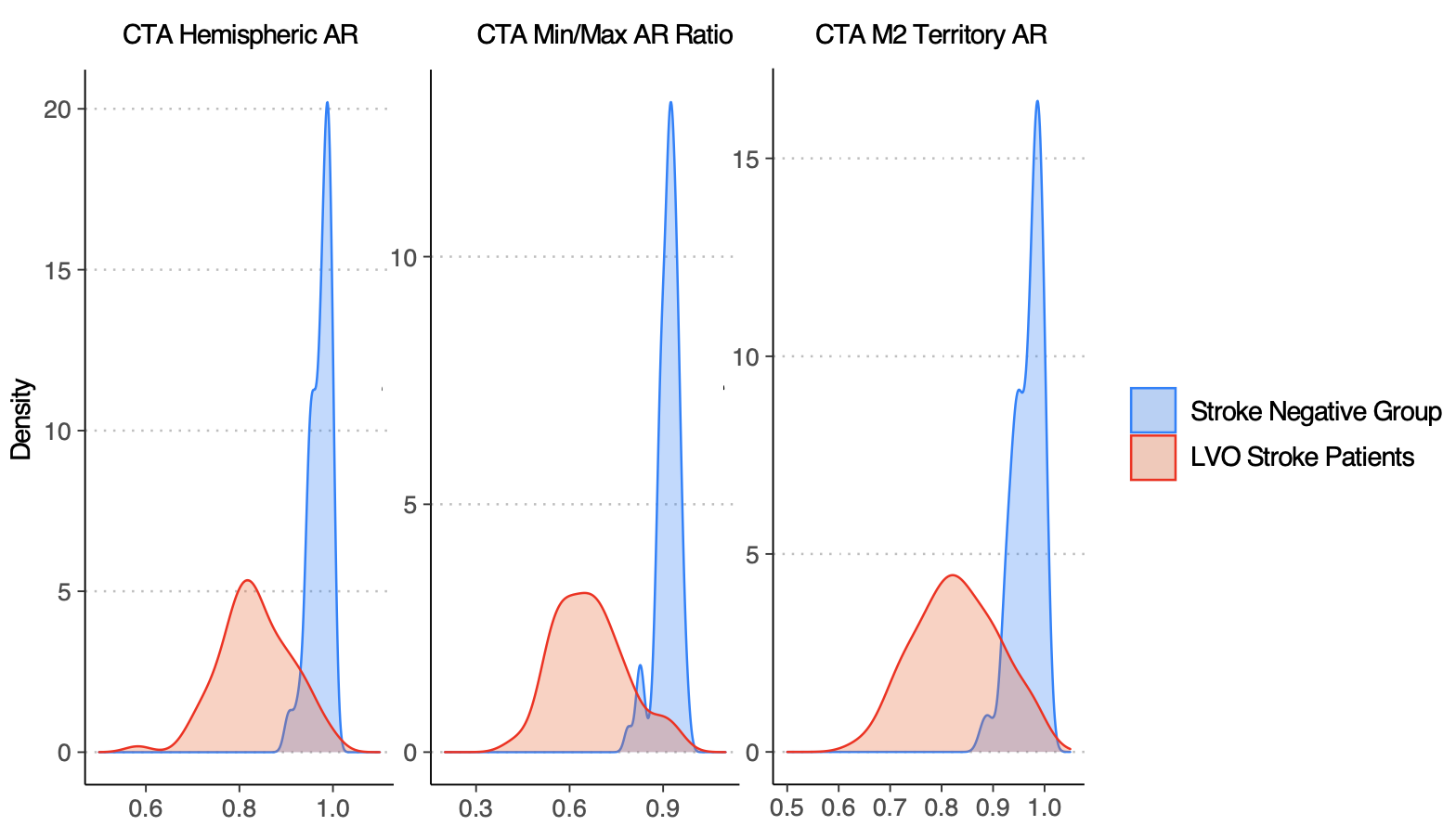
**

**Supplemental Figure II.** Density plot for the distribution of the indicated parameters in the stroke negative group (blue) and LVO stroke group (red). The x-axis displays the values of the respective parameters, the y-axis the kernel density estimate. LVO indicates, large vessel occlusion; CTA, CT Angiography, M2 segment of the middle cerebral artery.

| **Supplemental Table I.** Linear regression analysis for the association of white matter changes and automated CTA measurements (N=145) | | | |
| --- | --- | --- | --- |
|  | | | |
| **Independent variable** | **Beta (95% CI)** | | **P Value** |
| CTA Hemispheric AR | 0.061 | (-0.012-0.025) | 0.47 |
| CTA Min/Max AR Ratio | 0.019 | (-0.027-0.034) | 0.82 |
| CTA M2 Territory AR | 0.053 | (-0.013-0.025) | 0.53 |
| CTA ASPECTS M5 Region AR | 0.066 | (-0.006-0.014) | 0.43 |
| Univariate linear regression analyses were performed for the indicated imaging parameters as independent variables and the age-related white matter changes score (1) as dependent variable. CTA indicates CT angiography; AR, asymmetry ratio; ASPECTS, Alberta Stroke Program Early CT Score. P values <0.05 indicate statistical significance. | | | |

**Supplemental References:**1. Wahlund, L. O., et al. (2001). "A new rating scale for age-related white matter changes applicable to MRI and CT." Stroke 32(6): 1318-1322
